# SARS-CoV-2: Operating room management strategies and recommendations

**DOI:** 10.3389/fmed.2022.933799

**Published:** 2022-09-02

**Authors:** Wen-jun Zhang, Fei-long Zou, Dong-xia Hu, Hong-liang Luo, Li-dong Wu, Jia-ling Hu

**Affiliations:** ^1^Department of Rehabilitation Medicine, The Second Affiliated Hospital, Nanchang University, Nanchang, Jiangxi, China; ^2^Emergency Department, The Second Affiliated Hospital, Nanchang University, Nanchang, Jiangxi, China; ^3^Department of Gastrointestinal Surgery, The Second Affiliated Hospital, Nanchang University, Nanchang, Jiangxi, China

**Keywords:** SARS-CoV-2, operating room, management, recommendations, strategies

## Abstract

Since the outbreak of SARS-CoV-2/COVID-19 in Wuhan, China in 2019, it has rapidly spread to the world, and the number of infections has gradually increased. The hospitalization rate of patients has also gradually increased, which poses a huge challenge to hospitals and medical staff for patients with SARS-CoV-2 requiring surgical treatment. Therefore, avoiding cross-infection in the operating room is an important protective work. The operating room is an important department of the hospital, scientific and reasonable management is particularly important. Therefore, we have put forward corresponding suggestions and strategies for preoperative preparation and evaluation of patients, intraoperative management, postoperative terminal management, and protection of medical staff, and hope that these measures can better prevent and control the infection of SARS-CoV-2 in the operating room.

## Introduction

At the end of 2019, a kind of novel coronavirus pneumonia broke out in Wuhan city, Hubei province, China. The epidemic developed rapidly and spread to the whole country (1, 2). So far, the epidemic has spread all over the world and the number of diagnoses exceeds 500 million,^1^ and the global epidemic is very serious (3, 4). The WHO announced that the novel coronavirus pneumonia epidemic is a public health emergency of international concern (5, 6). The WHO initially named the virus 2019 novel coronavirus (2019-nCoV), later, the English name was revised to coronavirusdisease2019 (SARS-CoV-2) (7). For patients with SARS-CoV-2 who need emergency surgery, preventing intraoperative cross-infection is an extremely important task. Medical staff may be exposed to saliva droplets, blood, and secretions of patients with confirmed or suspected SARS-CoV-2 in their daily work, and the probability of exposure to high-risk groups is greatly increased (8, 9). Therefore, it is particularly important to increase the protection of medical staff. The operating room as an important department is also very easy to cause cross-infection in the hospital. Here, we refer to the prevention and control technology of the National Health Commission and SARS-CoV-2 diagnosis and treatment guidelines (10), combined with our hospital’s medical management experience, put forward SARS-CoV-2 patients’ emergency surgery operating room management strategies and recommendations, urgently formulated a SARS-CoV-2 patient surgical procedure, infection prevention, and control measures. We constantly improve, refine, and perfect the management and procedures of prevention and control in the operating room in our actual work. In this manuscript, we summarized the surgical procedures, infection prevention and control measures, and work experiences, and provided a reference for the first-line anti- SARS-CoV-2 workers.

## Screen and evaluate patients before surgery

According to the epidemiological characteristics of SARS-CoV-2, the incubation period is 14 days, and many asymptomatic infections may also become the source of infection (11). Therefore, it is important to understand the patient’s previous epidemiological history and clinical characteristics in detail. To grasp the timing of surgery can reduce the risk of surgical infection. In principle, it is not appropriate to perform elective or limited-term surgery for patients with SARS-CoV-2 (12, 13). For patients who must undergo elective surgery, such as tumors, it is recommended that patients must be isolated at home for 14 days. However, for some patients whose overall condition is poor, patients who are unable to carry out post-surgery after home isolation still need hospitalization adjustment and can be observed in the hospital for 14 days and given overall adjustment. In addition, nucleic acid testing is required before admission, and body temperature is checked three times a day. Surgery can only be performed when the patient is asymptomatic and the overall level of the body is assessed to be able to withstand surgery. Moreover, before the operation, anesthesiologists, nurses, and surgeons should communicate with each other for SARS-CoV-2 patients who may have serious life-threatening conditions, such as acute abdomen, major bleeding, emergency cesarean section, emergency abortion, emergency esophageal and tracheal foreign bodies, emergency surgery for brain trauma, and trauma (14–16). Before performing emergency surgery, the disease status of patients with SARS-CoV-2 should be understood in detail, such as body temperature, laboratory, and imaging examinations, and immediately report to the relevant departments of the hospital, and perform the surgery according to the surgical protection procedures for patients with SARS-CoV-2 (17). Furthermore, except for endotracheal intubation, patients should wear surgical masks throughout the procedure.

## Recommend operating room partition management

In the special period of SARS-CoV-2, different operations should be reasonably managed to distinguish between emergency surgery, limited-term surgery, and surgery for patients with suspected and confirmed SARS-CoV-2 (18).

a. Surgery within a limited period: The protective measures and levels of the surgical staff are the same as those for daily surgery. It is recommended to wear disposable anti-liquid leakage surgical gowns.
b. Emergency surgery: Emergency surgery should be performed in a relatively independent surgical area. Surgical staff should wear double-layer disposable hats, disposable liquid-proof surgical gowns, double-layer gloves, goggles/and protective screens, and shoe covers (wear medical protective masks (N95) if necessary). Before entering the operation room, close the laminar flow. After entering the operation room, all personnel should not leave the operating room. When the articles in the operating room are not enough or other special articles are needed, the mobile nurse can be responsible. After the operation, routine infectious surgery was performed.
c. Surgery for patients with suspected and confirmed SARS-CoV-2 should be performed in the negative pressure operating room (18). The negative pressure operating room should be equipped with independent material preparation and disinfection isolation treatment areas, with independent clean channels, patient channels, and dirt channels. SARS-CoV-2 sign is set at the entrance of the clean corridor, and clean areas and contaminated areas are strictly set up. Adjust the air purification system to negative pressure 1 h before the operation to keep the negative pressure value below −5 Pa. The warning sign “SARS-CoV-2” is hung outside the operating room, and the materials unrelated to the operation are removed from the operating room. If other articles are needed during the operation, the nursing personnel in the buffer front room are responsible for delivering the required articles to the operation room (19).

## Preparation and evaluation before surgery

**FIGURE 1:**
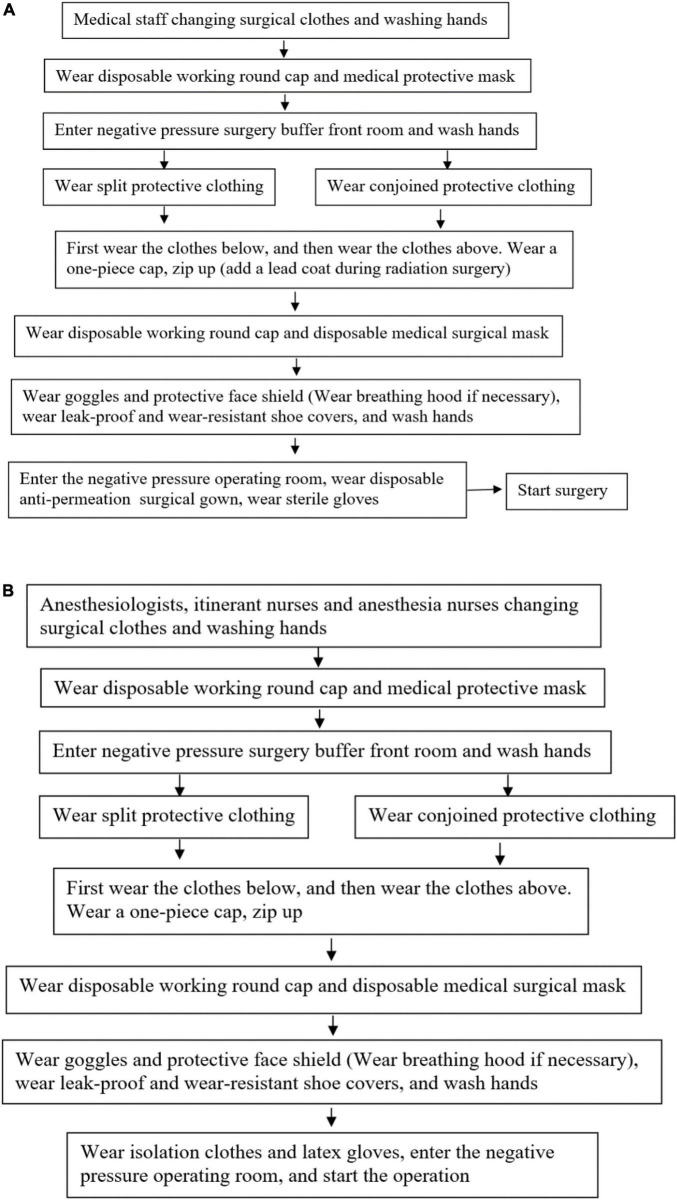
**(A)** Process of putting on medical protective articles for surgeons and instrument nurses in surgical operations on patients with confirmed or suspected SARS-CoV-2. **(B)** Process of putting on medical protective articles for the anesthesiologist, circuit nurse, and anesthetic nurse in surgical operations on patients with confirmed or suspected SARS-CoV-2.

Based on the epidemic situation, it is recommended that the relevant departments of the hospital, the anesthesiology department, and the operating room jointly establish a team of specialists to report and review the surgical personnel involved in patients with SARS-CoV-2, including surgeons, anesthesiologists, operating room nurses, circuit nurses, and assist the patient transfer nurses (19, 20). Importantly, medical personnel carrying out protective training consider the following aspects:

a. Preparation of articles before surgery: Surgical nurses and itinerant nurses prepare the corresponding articles for the operation according to the type of operation, and prepare the necessary articles for the operation as much as possible before the operation, including required equipment and accessories, sterile instrument bags, surgical instruments and dressings, medicines, disposable consumables, and high-value consumables, which can reduce the flow of personnel. Special needs to be prepared during the operation of the negative pressure smoking fog device. Surgery should be equipped with at least two sets of negative pressure aspirators, of which one set is for anesthesiologists only. After the patient enters the room, place a negative pressure suction tube on the patient’s head and face to minimize the spread of the patient’s respiratory secretions in the air. Additionally, inform the relevant departments of the hospital to make preparations, such as the disinfection supply center and the logistics office. The temporarily needed articles are delivered from the transfer window to the operating room, the principle is that only items can enter and not exit.
b. Protective measures for medical personnel (19, 21): Wear a mask (N95), wear protective clothing, disposable surgical gowns, goggles, protective face screen, long shoe covers, etc. It is an important measure for the medical staff participating in the operation to wear medical protective equipment to prevent cross infection, and the specific operation is shown in Figures 1A,B.^2^

c. After receiving the notification of confirmed or suspected SARS-CoV-2 patients requiring emergency surgery, the anesthesiologist, surgical nurse, and surgeon immediately communicate with each other and report to the hospital epidemic prevention working group according to the process. The nurse on duty immediately turns on the negative pressure equipment in the operating room, closes the buffer room, monitors the negative pressure value in the operating room (below −5Pa), and makes a record, and the “SARS-CoV-2”warning sign is hung outside the operating room (22).
d. Patient transfer: The patient is transported by the training medical staff and the rescue supplies needed are prepared during the transfer. Moreover, the patient needs to wear a disposable medical surgical mask during the transfer. The patient is transferred to the negative pressure operation room according to the designated transfer route of the isolated patient. Except for the transfer personnel, try to reduce the number of other personnel traveling, avoid stopping on the way, and prevent cross infection. All the preparatory work for the patient before the operation is completed in the negative pressure operating room.

## Intraoperative management

a. Intraoperative protection: In the operating room, except for the operating personnel, strictly restrict the entry of personnel not related to the operation. An additional roving nurse is assigned to the buffer zone to coordinate the preparation of articles in the operating room. Surgeons and nurses should wear double-layer latex gloves, it is recommended to use puncture-proof needles during the operation, and transfer sharp instruments to achieve non-contact transfer. Surgeons reasonably use electrosurgery and energy equipment to attract and expel smoke in time to avoid aerosol injury. Note that if the mask, goggles, or protective screen are contaminated by blood or body fluids, they should be replaced in time (21, 23).
b. Specimen treatment: Routine verification, collection, and registration of specimens; specimen bags are recommended to be double-sealed, and transfer boxes must be sealed. The itinerant nurse marks the “SARS-CoV-2” warning sign outside the surgical specimen information register, specimen bag, and transfer box, and hands it to the inspectors wearing medical protective clothing in the surgical buffer room for inspection.

## Postoperative management

**FIGURE 2:**
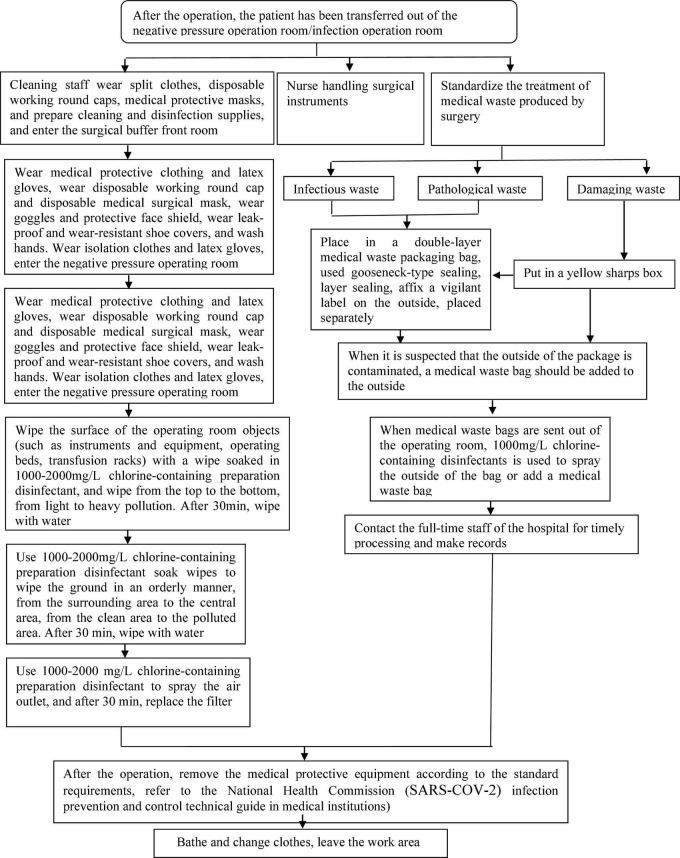
Final treatment of medical waste.

a. Patient management: After the operation, the patient was assessed to meet the conditions of coming out of the operating room. When the condition permits, patients should wear disposable medical surgical masks. Transfer medical staff carry transfer monitoring equipment, use special transfer beds and special channels, and send patients to isolation wards according to special routes. After handing over the patient, place contact items such as the transfer bed and monitoring equipment in the isolation treatment room for disinfection. After the transfer of the patient is completed, the medical protective articles are sequentially removed in the isolated area according to the specifications and placed in a medical waste container with the “SARS-CoV-2” logo.
b. Treatment of protective articles for medical staff: All medical staff involved in the operation take off protective articles and throw them into the designated medical waste container with the “SARS-CoV-2” logo. Protective clothing and other non-disposable articles are wrapped in medical waste bags and placed in designated areas for disposal. The specific order of taking off protective articles is taking off shoe covers → removing gloves → hand disinfection → taking off protective clothing → handing disinfection → removing goggles/protective face screen → handing disinfection → removing masks → handing disinfection → removing hat → handing disinfection → hot water bathing → changing personal clothes.
c. Surgery room treatment: Operating room should be fully disinfected according to the “special infection” process. Before disinfection, the precision instruments in the operating room should be protected with a protective sleeve to prevent corrosion. Turn off the laminar flow and supply air, use peracetic acid/hydrogen peroxide spray sterilizer to sterilize for 1–2 h, and then the operating room should be closed for at least 2 h, and then replace the return air and exhaust port filters and purify the internal filters of the unit, and then turn on laminar flow and ventilation. The ground with blood and body fluids scattered should be washed with 2,000–5,000 mg/L chlorine-containing preparation for 30 min, and then mop with clean water. Moreover, use 1,000–2,000 mg/L of chlorine-containing preparations for disinfection on the surfaces of equipment and operating tables for 10–30 min, and then wipe with clean water. Note that the surface of objects contaminated with blood and body fluids is directly treated with a chlorine-containing preparation of 2,000–5,000 mg/L. Finally, after disinfection, the infection control personnel should check and confirm that there is no source of infection before continuing to use. It is worth mentioning that the cleaning of the operating room should be done by the medical staff to assist the cleaning staff, and the cleaning staff should be given training and understood personal protection measures before cleaning.
d. Medical wastes treatment: Medical wastes should be placed in double-layer medical waste packaging bags, squeezing is strictly prohibited, gooseneck seals are used, layer sealing, and the “SARS-CoV-2” logo is affixed on the outside. It is recommended that the medical waste should be stored separately. Before leaving the operating room, spray 1,000 mg/L chlorine disinfectant on the surface of the medical waste packaging bag or add a layer of medical waste packaging bag on the outside. The flow chart of waste terminal treatment is shown in Figure 2.

e. Post-operation management of medical staff: All medical staff involved in the operation of confirmed or suspected SARS-CoV-2 patients are required to perform “medical observation” for 2 weeks. During the observation period, according to the clinical symptoms and signs of SARS-CoV-2, monitor the body temperature regularly and report to the competent department. If abnormalities occur during the observation period, seek medical treatment in time.

## Summary

Currently, the situation of SARS-CoV-2 is still serious and there are some asymptomatic people (24, 25). The hospital is the center of SARS-CoV-2 patient treatment. Therefore, the epidemic brings great challenges to the hospital. Studies have shown that patients with SARS-CoV-2 may have nosocomial cross infection early (26). The operating room is an important department of the hospital, and there is a high risk of cross infection. By conducting SARS-CoV-2 investigations on surgical patients and implementing surgical zoning management, using negative pressure operating rooms for suspected and confirmed SARS-CoV-2 patients. Scientifically and rationally perform staffing, articles management, effective surgical protection, and strict implementation of the disinfection and isolation system after surgery. It is of great significance to form a control and management plan for the operating room under the new coronavirus pneumonia epidemic, to ensure the safety of patients and medical staff. All medical personnel must strictly follow the specifications to ensure the normal operation of the operating room and avoid cross-infection in the hospital.

## Data availability statement

The original contributions presented in this study are included in the article/supplementary material, further inquiries can be directed to the corresponding author.

## Author contributions

All authors listed have made a substantial, direct, and intellectual contribution to the work, and approved it for publication.

## Footnotes

1
https://covid19.who.int/

2
https://www.nejm.org/doi/full/10.1056/NEJMvcm2014809?query=C19&cid=DM92284_NEJM_Registered_Users_and_InActive&bid=201603318
